# Lipid-lowering drug adherence and combination therapy effects on gastrointestinal cancer in patients with dyslipidemia without diabetes: a retrospective cohort study in South Korea

**DOI:** 10.1186/s12885-022-09250-8

**Published:** 2022-02-08

**Authors:** Kyu-Tae Han, Seungju Kim

**Affiliations:** 1 Division of Cancer Control & Policy, National Cancer Control Institute, National Cancer Center, Goyang, Republic of Korea; 2 Department of Nursing, College of Nursing, The Catholic University of Korea, 222, Banpo-daero, Seocho-gu, Seoul, 06591 Republic of Korea

**Keywords:** Lipid-lowering agents, Combination therapy, Medication possession ratio, Gastrointestinal cancer, Statins

## Abstract

**Background:**

In aging populations, the number of people with high cholesterol levels is increasing. Appropriate management of high cholesterol levels with drugs such as statins may prevent secondary diseases. Despite many studies on the effects of statins on various types of cancer, the effectiveness of lipid-lowering therapy in preventing cancer remains controversial. This study aimed to evaluate its long-term effect on developing gastrointestinal (GI) cancer in patients with dyslipidemia.

**Methods:**

This study used the National Health Insurance Sampling (NHIS) cohort data (2002–2015), which included patients with dyslipidemia without diabetes, and measured patients’ adherence to lipid-lowering therapy using the medication possession ratio. We used the Cox proportional hazard ratio (HR) to identify the association between the continuity of lipid-lowering therapy and the risk of GI cancer. We also evaluated the association between a combination of lipid-lowering drugs and a reduced risk of GI cancer.

**Results:**

A total of 49,351 patients were diagnosed with dyslipidemia, of which 579 were diagnosed with GI cancer. Patients with higher adherence to lipid-lowering therapy had a significantly reduced risk of GI cancer compared to patients without drugs, and high adherence was associated with a reduced incidence of all types of GI cancer. Specifically, the combination of statins and ezetimibe or fibrates appears to reduce GI cancer risk effectively. Overall, the continuity of lipid-lowering therapy had a protective effect on GI cancer in middle-aged and elderly patients with dyslipidemia compared to non-users.

**Conclusions:**

Our findings suggest that the continuity of lipid-lowering therapy is vital in patients with dyslipidemia. In addition, for individuals vulnerable to GI cancer, combination therapy may be associated with more effective protection against GI cancer. Healthcare providers need patient education and monitoring to improve drug adherence in patients with dyslipidemia.

**Supplementary Information:**

The online version contains supplementary material available at 10.1186/s12885-022-09250-8.

## Background

Unhealthy lifestyles with poor diet and insufficient exercise increase vulnerability to diseases, leading to increased morbidity and mortality from chronic diseases in aging populations [1, 2]. According to the disease burden in 2015, 71.3% of global deaths were due to non-communicable diseases, followed by 17.9 million cardiovascular disease (CVD)-related deaths and 8.8 million cancer deaths [3]. CVD and cancer, which are closely associated with lifestyle behaviors, share common risk factors [4], including dyslipidemia; therefore, the effective management of these risk factors may impact both CVD and cancer.

Dyslipidemia is a risk factor for various diseases, including CVD, and high serum cholesterol is frequently associated with an increased risk of esophageal and colorectal cancer (CRC) [5, 6] and cancer progression [7, 8]. A Korean study found that thyroid cancer patients with dyslipidemia have an increased risk of secondary cancers [9]; however, other studies have shown that patients with dyslipidemia have a decreased risk of cancer, including breast cancer [10, 11]. Abnormal cholesterol levels may influence cancer incidence considering these studies, and cholesterol treatment may affect cancer development.

According to the 2013 American College of Cardiology or American Heart Association guidelines, cholesterol-lowering medications are recommended to reduce CVD risk based on the target cholesterol levels [12]. Following these guidelines, many patients with dyslipidemia are treated with statins, and positive outcomes have been reported due to drug use. In patients with CVD risk factors, statins decrease the risk and improve the survival rate [13], and serum cholesterol levels in patients taking statins are inversely correlated with cancer risk [14]. At sufficiently high doses, statin use is associated with a reduced incidence of cancer [15] and a reduced rate of cancer-related mortality compared to non-statin users [16]. Combination therapy of ezetimibe or fibrates with a statin was associated with lower serum cholesterol levels and reduced CVD risk compared to monotherapy [17, 18]. In contrast, meta-analysis studies found that statin use did not reduce the incidence of cancer [19, 20]. Additionally, in clinical studies, the combination of simvastatin and ezetimibe was associated with reduced serum cholesterol levels but did not positively affect patient outcomes [21]. Although these results suggest the importance of taking statins to prevent cancer development, evidence for the effect of continued use and combination of lipid-lowering therapy on specific cancers is lacking.

Gastrointestinal (GI) cancer is one of the most common cancers in Korea [22], and the long-term use of statins may be beneficial for cancer prevention. Clinical guidelines for dyslipidemia suggest monotherapy or combination therapy depending on the patient’s condition, suggesting that drug use may have different effects on the development of gastrointestinal cancer.

In this study, we evaluated the effect of adherence to lipid-lowering therapy and the effect of this combination on the development of GI cancer. We evaluated the effects of adherence to lipid-lowering therapy for every GI cancer until the onset of cancer and analyzed each cancer type. Subsequently, we evaluated the association between a combination of lipid-lowering therapy and the development of GI cancer. In addition, these effects may differ according to age, and to evaluate the more vulnerable groups, we evaluated the effects of continuity of lipid-lowering therapy on the development of cancer according to age in a subgroup analysis.

## Materials and methods

### Database and data collection

This study used the National Health Insurance Sampling cohort data from 2002 to 2015. This dataset included the baseline population of 1,025,340 randomly selected participants, representing 2.2% of the total eligible Korean population in 2002 [23]. We included demographic information, treatment data, death date, and hospital characteristics. Medical data for all subjects were included as part of their insurance claims and included diagnosis, comorbidities, medications, dates of visits, and cost.

During the study period, a total of 124,218 patients were diagnosed with dyslipidemia as the major diagnosis according to the International Classification of Diseases (ICD-10 code: E78). First, we excluded patients diagnosed with dyslipidemia between 2002 and 2003 to reduce the time bias for GI cancer development [24] and defined patients diagnosed from 2004 as newly diagnosed patients. Second, we considered other chronic diseases that can affect cancer incidence [25], and excluded patients diagnosed with diabetes, which is associated with the development of GI cancer. Third, we excluded patients younger than 30 years or older than 75 and those diagnosed with carcinoma other than GI cancer. To reduce time-related errors in cancer incidence, we excluded patients who had previously been diagnosed with GI cancer or developed GI cancer within 6 months of diagnosis based on the date of diagnosis of dyslipidemia. Fourth, prescribing data and patient data were merged to exclude patients who received a prescription for dyslipidemia in the previous year for dyslipidemia diagnosis on the first drug prescription date for each patient during the study period. In addition, a follow-up period of at least 6 months from the date of diagnosis of dyslipidemia was considered, and patients with a short follow-up period were excluded. Finally, patients with missing variables, such as income, were excluded. After these exclusions, 49,351 patients with dyslipidemia were included in the study.

### Variables

We evaluated the adherence to lipid-lowering therapy and statin combination therapy in this study. For adherence, we measured the medication possession ratio (MPR), the number of days that lipid-lowering therapy was supplied to each patient for each year of the study. Lipid-lowering therapy drugs were included according to the WHO ATC code: statin (C10AA01–08), fibrates (C10AB02–05, 11), bile acid sequestrants (C10AC01), ezetimibe (C10AX09), and combinations of various lipid-modifying agents (statin + ezetimibe: C10BA02, 05, 06; statin + fibrates: C10BA03) (Supplementary Table a). If the year the drug was first prescribed and the year of diagnosis was the same, but the dates were different, the date of diagnosis of dyslipidemia was defined based on the previous date. We used the total number of days the patient was prescribed lipid-lowering therapy each year, modified it so that it did not exceed 365 days, and divided that number by 365 days to calculate the annual MPR [26]. In cancer patients, the average MPR was calculated from the diagnosis date of dyslipidemia to cancer diagnosis and to the end of the study or death date for other patients. We classified MPR by quartiles (< 25, < 50, < 75%, ≥75). According to the guidelines for dyslipidemia in Korea, statin monotherapy is recommended as the primary therapy, and if the target serum cholesterol level is not reached, other lipid-lowering therapies are recommended [27]. We included all lipid-lowering drugs prescribed to patients during the study period and classified them into six categories: statin, fibrates or bile acid sequestrants, statin and ezetimibe, statin and fibrates, statin and fibrates or ezetimibe, and non-user.

The outcome variable was GI cancer, and cancer incidence was measured based on the ICD 10 code as follows: esophagus and stomach (C15, C16), colorectal (C18, C19, C20, C21), and liver, bile ducts, and pancreas (C22, C23, C24, C25). Cancer patients were defined as those diagnosed with cancer for the first time after a dyslipidemia diagnosis; therefore, we determined the diagnosis date of cancer was during the study period. Since the continuous management of dyslipidemia can be related to the treatment hospital, we considered the hospital where the patients were mainly treated as a variable. First, we calculated the number of visits to all medical institutions where patients were treated for dyslipidemia during the study period. Subsequently, the type of hospital with the most visits was calculated by dividing the number of visits to each institution by the total number of hospital visits, and the primary medical institution for each patient was classified into a community health center, clinic hospital, general hospital, or tertiary hospital.

Drugs other than lipid-lowering drugs that may affect the development of GI cancer include aspirin and metformin [28, 29]. Similar to lipid-lowering drugs, the annual MPR of the patients who were prescribed the drug during the study period was calculated, and the MPR of each drug ranges from 0 to 100. Patient demographic data included sex (male, female), age (30–44, 45–59, 60–75 years), residential area (capital area, metropolitan, other), insurance (Medicaid, self-employed, employee), and income (low, low-moderate, moderate-high, high). The average body mass index (BMI) data were classified based on Asian subjects: under-weight (< 18.5 kg/m^2^), normal range (18.5–22.9 kg/m^2^), overweight (23–24.9 kg/m^2^), obese I (25–29.9 kg/m^2^), obese II (≥30 kg/m^2^) [30]. The patient’s severity measured by the Charlson Comorbidity Index (CCI) and the year of dyslipidemia diagnosis (2004–2007, 2008–2011, 2012–2015) were also included.

### Ethical consideration

This study was conducted using secondary data, which are public data, and individuals were encrypted and could not be identified. According to the Bioethics and Safety Act, this study presents only the minimum risk. This study was approved by the Institutional Review Board of the Catholic University of Korea (IRB number: MC21ENSI0043).

### Statistical analysis

The distribution of each categorical variable was examined by analyzing the frequency and percentage using the χ^2^ test. For continuous variables, t-tests were performed to compare the means and standard deviations. We used the Cox proportional hazard ratio (HR) to identify the association between adherence to lipid-lowering therapy and the GI cancer risk. The start date was the first day of diagnosing dyslipidemia or prescribed lipid-lowering therapy, and the end date was the date of GI cancer diagnosis, death, or the end of the study. All variables were entered simultaneously into the fully adjusted model. We evaluated all incidences of GI cancer, specific cancers of the esophagus and stomach, colorectal cancers, and cancers of the liver, bile ducts, and pancreas. In addition, we conducted an analysis to evaluate the relationship between statin combination therapy and GI cancer. We conducted subgroup analysis by age to evaluate the associations between adherence to lipid-lowering therapy and GI cancer. All statistical analyses were performed using SAS statistical software (SAS Institute, Cary, NC, USA). Statistical significance was set at *p* < 0.05.

## Results

Table 1 shows the general characteristics of patients with dyslipidemia. A total of 49,351 patients were diagnosed with dyslipidemia, of which 579 (1.2%) were diagnosed with GI cancer. Among these, 30.1% did not receive lipid-lowering drugs, and 1.1% (*n* = 161) developed GI cancer. The incidence of GI cancer was highest in patients with poor adherence (MPR < 25%, *n* = 205, 1.3%) and lowest in patients with high adherence (MPR ≥75, *n* = 40, 1.0%), but the difference was not statistically significant. The average drug adherence was 8.89% for aspirin and 0.42% for metformin. Most patients received treatment at a clinic, and the proportion of women with dyslipidemia was high. The mean follow-up period was 64.2 months.

**Table 1:** General characteristics of patients with dyslipidemia(*n* = 49,351)

	Gastrointestinal cancer				Total		***p***-value
	Yes		No				
**Adherence to lipid-lowering medications**							
non-user	161	(1.1)	14,702	(98.9)	14,863	(30.1)	0.2915
MPR < 25%	205	(1.3)	15,499	(98.7)	15,704	(31.8)	
MPR < 50%	96	(1.2)	8064	(98.8)	8160	(16.5)	
MPR < 75%	77	(1.2)	6404	(98.8)	6481	(13.1)	
MPR ≥ 75%	40	(1.0)	4103	(99.0)	4143	(8.4)	
**Drug adherence (MPR, %)**							
Aspirin	11.26	± 24.25	8.86	± 23.23	8.89	± 23.24	0.0182
Metformin	0.40	± 4.52	0.42	± 5.33	0.42	± 5.32	0.9211
**Primary medical institution for treatment of dyslipidemia**							
Community health center	32	(1.9)	1652	(98.1)	1684	(3.4)	0.0005
clinic	360	(1.1)	33,217	(98.9)	33,577	(68.0)	
Hospital	60	(1.2)	4931	(98.8)	4991	(10.1)	
General hospital	88	(1.3)	6940	(98.7)	7028	(14.2)	
Tertiary hospital	39	(1.9)	2032	(98.1)	2071	(4.2)	
**CCI**	3.02	± 1.77	2.42	± 1.61	2.43	± 1.61	< 0.0001
**Sex**							
Male	314	(1.4)	21,592	(98.6)	21,906	(44.4)	< 0.0001
Female	265	(1.0)	27,180	(99.0)	27,445	(55.6)	
**Age**							
30–44	51	(0.4)	12,170	(99.6)	12,221	(24.8)	< 0.0001
45–59	245	(1.0)	24,948	(99.0)	25,193	(51.0)	
60–75	283	(2.4)	11,654	(97.6)	11,937	(24.2)	
**BMI**							
< 18.5	8	(1.1)	717	(98.9)	725	(1.5)	0.4253
18.5–22.9	185	(1.1)	16,304	(98.9)	16,489	(33.4)	
23–24.9	183	(1.3)	13,684	(98.7)	13,867	(28.1)	
25–29.9	186	(1.1)	16,350	(98.9)	16,536	(33.5)	
≥ 30	17	(1.0)	1717	(99.0)	1734	(3.5)	
**Residual area**							
Capital area	266	(1.2)	21,255	(98.8)	21,521	(43.6)	0.2325
Metropolitan	139	(1.0)	13,236	(99.0)	13,375	(27.1)	
Other	174	(1.2)	14,281	(98.8)	14,455	(29.3)	
**Income**							
Low	131	(1.2)	11,114	(98.8)	11,245	(22.8)	0.7941
Low-moderate	132	(1.1)	11,814	(98.9)	11,946	(24.2)	
Moderate-high	129	(1.2)	10,921	(98.8)	11,050	(22.4)	
High	187	(1.2)	14,923	(98.8)	15,110	(30.6)	
**Insurance**							
Medicaid	16	(1.6)	1012	(98.4)	1028	(2.1)	0.0183
Self-Employed	217	(1.3)	15,932	(98.7)	16,149	(32.7)	
Employees	346	(1.1)	31,828	(98.9)	32,174	(65.2)	
**Year of diagnosis**							
2004–2007	272	(2.4)	10,888	(97.6)	11,160	(22.6)	< 0.0001
2008–2011	241	(1.3)	18,703	(98.7)	18,944	(38.4)	
2012–2015	66	(0.3)	19,181	(99.7)	19,247	(39.0)	
**Mean follow-up periods (month)**	51.20	± 30.90	64.39	± 37.70	64.24	± 37.65	< 0.0001
**total**	579	(1.2)	48,772	(98.8)	49,351	(100.0)	

*MPR* medication possession ratio, *CCI* Charlson Comorbidity Index

Table 2 shows the results of the association between adherence to lipid-lowering therapy and GI cancer. A low MPR (< 25%) increased the risk of GI cancer compared to non-users, but this difference was not statistically significant. Patients with higher MPR (< 50% HR: 0.741, 95% CI: 0.574–0.958; < 75% HR: 0.636, 95% CI: 0.480–0.842; ≥75 HR: 0.438, 95% CI: 0.305–0.629) had a significantly reduced risk of GI cancer compared to non-users. Higher adherence to aspirin and metformin was associated with a reduced risk of GI cancer, but only aspirin was statistically significant (HR, 0.995; 95% CI, 0.998). The risk of GI cancer increased with age (age 45–60 HR: 2.612, 95% CI: 1.906–3.579; age 61–75 HR: 5.656, 95% CI: 4.000–7.998).

**Table 2:** The association between continuity use of lipid-lowering therapy and GI cancer

	Gastrointestinal cancer		
	HR	95% CI	
**Adherence to lipid-lowering therapy**			
non-user	Ref	–	–
MPR < 25%	1.029	0.835	1.268
MPR < 50%	0.741	0.574	0.958
MPR < 75%	0.636	0.480	0.842
MPR ≥ 75%	0.438	0.305	0.629
**Drug adherence (MPR, %)**			
Aspirin	0.995	0.991	0.998
Metformin	0.996	0.980	1.012
**Primary medical institution for treatment of dyslipidemia**			
Community health center	0.752	0.468	1.209
clinic	0.711	0.506	0.999
Hospital	0.868	0.573	1.314
General hospital	0.766	0.523	1.121
Tertiary hospital	Ref	–	–
**CCI**	1.193	1.127	1.263
**Sex**			
Male	1.874	1.582	2.219
Female	Ref	–	–
**Age**			
30–44	Ref	–	–
45–59	2.612	1.906	3.579
60–75	5.656	4.000	7.998
**BMI**			
< 18.5	1.009	0.496	2.051
18.5–22.9	Ref	–	–
23–24.9	1.036	0.844	1.272
25–29.9	0.888	0.723	1.091
≥ 30	0.953	0.578	1.571
**Residual area**			
Capital area	1.121	0.924	1.360
Metropolitan	0.937	0.749	1.172
Other	Ref	–	–
**Income**			
Low	1.085	0.857	1.373
Low-moderate	1.049	0.838	1.313
Moderate-high	1.073	0.856	1.344
High	Ref	–	–
**Insurance**			
Medicaid	1.410	0.832	2.391
Self-Employed	1.107	0.932	1.315
Employees	Ref	–	–
**Year of diagnosis**			
2004–2007	Ref	–	–
2008–2011	0.688	0.556	0.851
2012–2015	0.631	0.458	0.867

*MPR* medication possession ratio, *CCI* Charlson Comorbidity Index, *HR* Hazard Ratio, *95% CI* 95% confidence interval

**Fig. 1:**
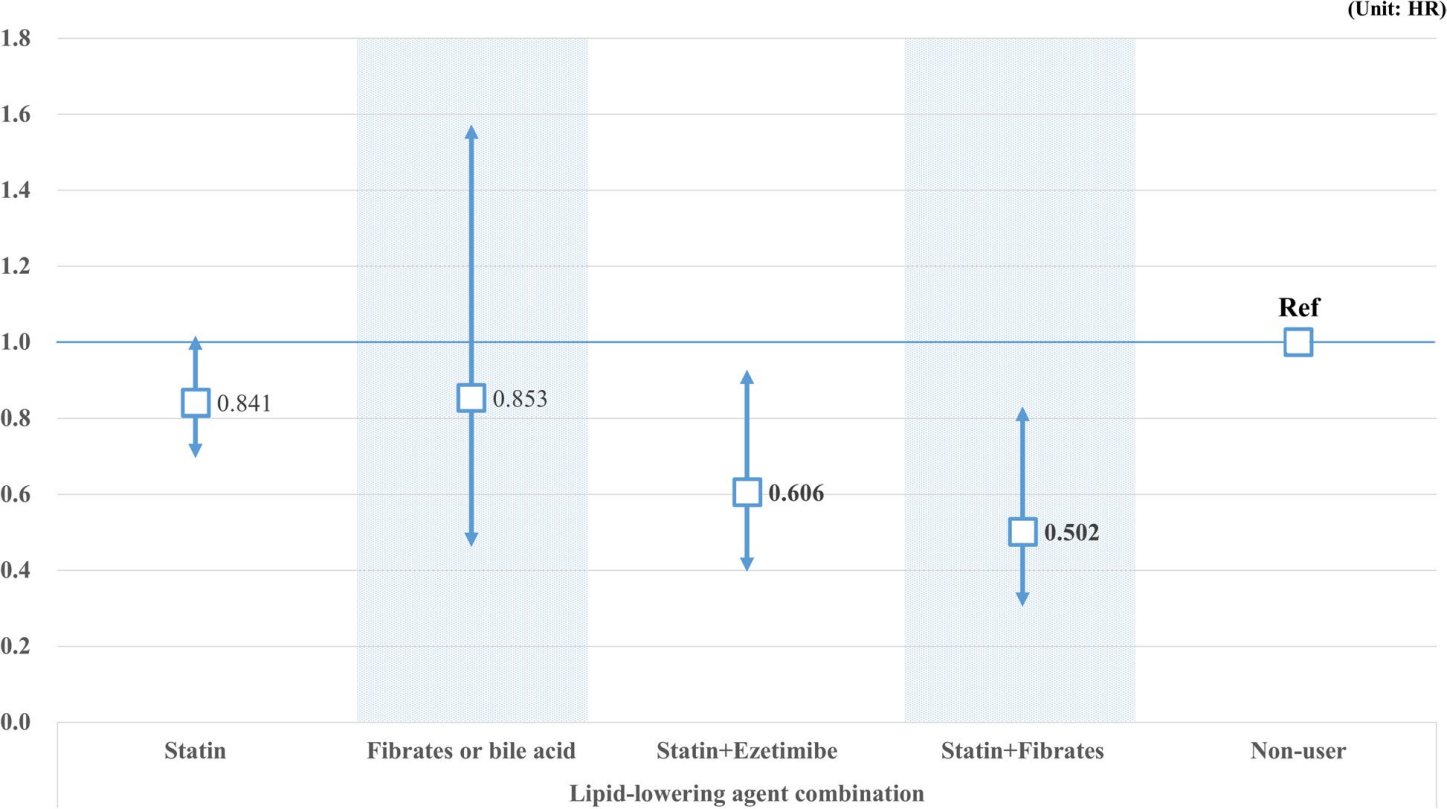
The relationship between monotherapy or combination prescription of lipid-lowering drugs and the incidence of GI cancer

**Fig. 2:**
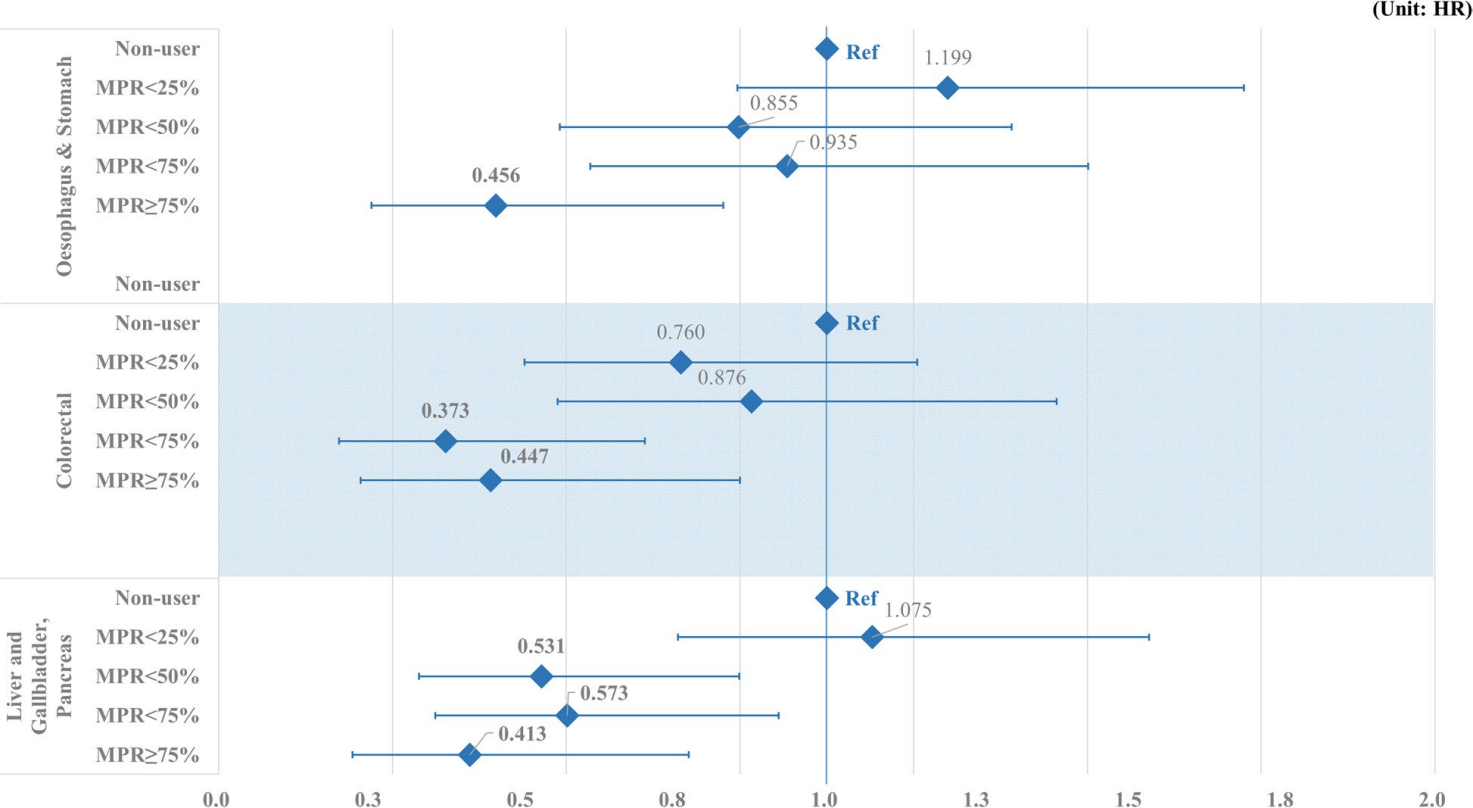
The relationship between adherence to the use of statins and the incidence of specific cancers

Appendix Table b shows patients who were prescribed lipid-lowering drugs. Most patients were prescribed statins, and 13.2% of patients were prescribed a combination of statins and ezetimibe or fibrate. Figure 1 shows the association between the combination of lipid-lowering therapy and the risk of GI cancer. Statins or fibrates and bile acid monotherapy were associated with a reduced risk of GI cancer, but the difference was not statistically significant. Combination therapy with a statin and ezetimibe (HR, 0.606; 95% CI: 0.396–0.927) or fibrate (HR: 0.502, 95% CI: 0.304–0.831) was associated with a statistically significant reduction in the gastrointestinal cancer risk. Figure 2 shows the effects of lipid-lowering therapy on specific cancers of the esophagus, stomach, colon and rectum, liver, bile ducts, and pancreas. Patients with an MPR < 75% had a significantly reduced risk of esophageal or stomach cancer than non-users (HR: 0.456, 95% CI: 0.251–0.830). Concerning colorectal cancer, a high MPR was associated with a decreased risk of cancer, but it was statistically significant only for an MPR > 50% (< 75% HR: 0.373, 95% CI: 0.198–0.701; ≥75 HR: 0.447, 95% CI: 0.233–0.857). A high MPR was significantly associated with a reduced risk of cancers of the liver and gallbladder, and the pancreas (< 50% HR, 0.531; 95% CI, 0.329–0.856; < 75% HR: 0.573, 95% CI: 0.356–0.921; ≥75 HR: 0.413, 95% CI: 0.220–0.773).

Table 3 shows the results of the subgroup analysis of adherence to lipid-lowering therapy for GI cancer by age. At age 44 years and younger, a high MPR was associated with a decreased risk of GI cancer, but this was not statistically significant. In patients aged 45–59 years, the trend was similar but was statistically significant only for an MPR ≥ 50% (< 75% HR, 0.471; 95% CI, 0.294–0.753; ≥75 HR, 0.362; 95% CI, 0.192–0.681). In patients aged 60 years and older, a high MPR significantly reduced the risk of GI cancer compared to non-users (> 75 HR: 0.469; 95% CI, 0.295–0.744).

**Table 3:** Results of subgroup analysis of the association between adherence to statin use for GI cancer by age

	30–44			45 ~ 59			60 ~ 75		
	HR	95% CI		HR	95% CI		HR	95% CI	
**Adherence Level**									
non-user	Ref	–	–	Ref	–	–	Ref	–	–
MPR < 25%	1.081	0.59	1.981	0.967	0.705	1.325	1.067	0.779	1.462
MPR < 50%	0.501	0.169	1.486	0.762	0.523	1.111	0.727	0.498	1.063
MPR < 75%	0.363	0.079	1.668	0.471	0.294	0.753	0.761	0.524	1.104
MPR ≥ 75%	0.354	0.042	2.961	0.362	0.192	0.681	0.469	0.295	0.744

*MPR* medication possession ratio, *HR* Hazard Ratio, *95% CI* 95% confidence interval

## Discussion

Dyslipidemia is a significant risk factor for cancer and other diseases, and continued management with statins may have protective effects against the risk of secondary disease. This study found that high adherence to lipid-lowering therapy was associated with a decreased risk of GI cancer. Specifically, statin combination therapy appears to be more effective than statin monotherapy. As MPR increased, the risk of GI cancer gradually decreased, with significant protective effects against the esophagus and stomach, colorectal cancers, and cancers of the liver, bile ducts, and pancreas. The protective effect of lipid-lowering drugs against cancer appears to be crucial in middle-aged and older adults.

It was first thought that statins primarily lower serum lipid levels when in actuality, they affect many metabolic pathways and organ systems and have a positive effect on several diseases, and reduce low-density lipoprotein cholesterol [31]. This effect is known as the pleiotropic effect of statins, and statins may be involved in membrane receptor signaling, such as cell proliferation or angiogenesis, by inhibiting the mevalonate pathway [32]. Preclinical and clinical studies have demonstrated that statins have anti-tumor effects, inhibiting tumor development and growth [33]. The ability of statins to increase apoptosis and decrease the proliferation of specific cancer cells may explain their ability to reduce the cancer risk [32, 33]. Our findings also support the anti-tumor effects of statins and indicate that the sustained use of statins is crucial. Specifically, although not statistically significant, the risk of GI cancer was increased in patients with an MPR of less than 25% compared to non-users, suggesting the importance of continuous drug adherence. Low drug adherence may be related to poor communication with patients, and healthcare providers should educate patients that continuous use of statins is effective in lowering cholesterol and the prevention of secondary diseases such as GI cancer.

In addition, this study found that increased adherence to aspirin was associated with a reduced risk of GI cancer, similar to previous studies, suggesting that long-term aspirin use was associated with a protective effect against cancer, especially gastrointestinal tract tumors [28]. However, since long-term use of aspirin is also associated with an increased risk of gastrointestinal bleeding, it should be carefully considered [34], and whether it is used in combination with lipid-lowering agents will require further studies.

Several studies have shown that combination lipid-lowering therapy with statins is more effective than monotherapy by increasing the therapeutic effect [17, 18]. This study also found that combination therapy had a protective effect against GI cancer, particularly the combination of a statin with ezetimibe or fibrates. Ezetimibe is known to reduce cholesterol absorption from the gastrointestinal tract, and combination therapy reduced the risk of CVD compared to monotherapy [35]; however, not all studies found a positive effect on patient outcomes [21]. A meta-analysis found that fibrate-statin combination therapy effectively controlled serum cholesterol levels compared to fibrate monotherapy and increased the risk of adverse events [36]. These results suggest that combination therapy may be more effective than monotherapy, but careful drug selection is necessary to consider the patient’s condition and side effects.

Long-term use of statins reduced the risk of GI cancer, but only some MPRs were statistically significant, depending on the type of GI cancer. Previous studies on the effect of statins on GI cancers have shown no effect on stomach cancer, a mixed effect on colorectal cancer, and a reduction in pancreatic and liver cancer [37]. A study found that statin use was associated with a reduced risk of hepatocellular carcinoma, and atorvastatin and simvastatin were associated with a reduced risk of liver cancer, but this was not statistically significant [38]. Our study confirms that continued use of lipid-lowering therapy reduces the risk of GI cancers, suggesting that long-term statin therapy may benefit individuals at risk for GI cancers.

Age had a significant effect on lipid-lowering therapy and GI cancer. In general, patients showing high adherence to lipid-lowering therapy had a reduced risk of GI cancer. While this reduced risk was not statistically significant in the younger age group, it was significant in patients aged above 45 years. As adherence increased, the protective effect against GI cancer gradually increased; therefore, in middle-aged and elderly patients with dyslipidemia, long-term statin use could reduce the incidence of GI cancer and is most effective when adherence is greater than 75%. As older people become more susceptible to various diseases as they age, health care providers should regularly monitor their patients’ drug intake and educate them about the importance of continuing their statins.

Despite the many studies on the effects of lipid-lowering therapy on various types of cancer, the use of statins to prevent cancer remains controversial. Since we studied a representative population, our findings provide adequate evidence supporting the value of long-term use of lipid-lowering therapy. We provide evidence that continued lipid-lowering therapy effectively reduces GI cancers and that there should be appropriate monitoring of therapy for older, more vulnerable patients.

Nonetheless, our study has some limitations. First, since we did not include clinical data, we do not know the effect of the long-term use of lipid-lowering therapy on cholesterol levels or its association with cancer development. Therefore, further studies are needed to determine whether changes in cholesterol levels resulting from the continued use of drugs affect the incidence of cancer. Second, we only evaluated the incidence of GI cancers; further research is needed to determine the effect of lipid-lowering therapy on other types of cancer. Finally, although we excluded patients with diabetes and adjusted for BMI, other factors, including diet and lifestyle, were not included in this study and may have affected the outcome.

## Conclusions

This study found that long-term use of lipid-lowering therapy protects against the development of GI cancer. Specifically, the preventive effect of GI cancer was significant in combination therapy, and the continuous use of lipid-lowering drugs in middle-aged and older adults was associated with the protective effect of GI. Therefore, health care providers need to educate patients with dyslipidemia about the protective effects of statins against GI cancer, and monitoring the continued use of statins is essential.

## Supplementary Information

**Additional file 1: Supplementary Table a.** List of drugs included in the study. **Supplementary Table b.** Patients who have been prescribed lipid-lowering drugs.

## Data Availability

This data is public data and can be used with permission in accordance with the regulations of NHIS (https://nhiss.nhis.or.kr/bd/ab/bdaba000eng.do;jsessionid=MzhEE1iiO4JuhlK5JHhC2vfyEXsi76ZMdnLpiqdqPcRPGgoKJ1OeJI2aysgz6LyX.primrose22_servlet_engine10).

## Abbreviations

CVD: Cardiovascular disease
GI: Gastrointestinal
ICD: International Classification of Diseases
MPR: Medication possession ratio
DDDs: Defined daily doses
BMI: Body mass index
CCI: Charlson Comorbidity Index
