# Supplementary material for: Lipid-lowering drug adherence and combination therapy effects on gastrointestinal cancer in patients with dyslipidemia without diabetes: a retrospective cohort study in South Korea
Source: BMC Cancer. 2022 Feb 8;22:156. doi: 10.1186/s12885-022-09250-8 (PMC8826710; doi:10.1186/s12885-022-09250-8)
Supplement: Supplementary file 1 — Additional file 1: Supplementary Table a. List of drugs included in the study. Supplementary Table b. Patients who have been prescribed lipid-lowering drugs. [file 12885_2022_9250_MOESM1_ESM.docx]

Appendix

| **Supplementary Table a. List of drugs included in the study** | | |
| --- | --- | --- |
|  | ATC code | Name |
| Statin | C10AA01 | simvastatin |
|  | C10AA02 | lovastatin |
|  | C10AA03 | pravastatin |
|  | C10AA04 | fluvastatin |
|  | C10AA05 | atorvastatin |
|  | C10AA07 | rosuvastatin |
|  | C10AA08 | pitavastatin |
| Fibrates | C10AB02 | bezafibrate |
|  | C10AB04 | gemfibrozil |
|  | C10AB05 | fenofibrate |
|  | C10AB11 | choline fenofibrate |
| Bile acid sequestrants | C10AC01 | colestyramine |
| Other lipid modifying agents | C10AX09 | ezetimibe |
| Combinations of  various lipid modifying agents | C10BA02 | simvastatin and ezetimibe |
|  | C10BA03 | pravastatin and fenofibrate |
|  | C10BA05 | atorvastatin and ezetimibe |
|  | C10BA06 | rosuvastatin and ezetimibe |

| **Supplementary Table b. Patients who have been prescribed lipid-lowering drugs** | | | | | | | |  |
| --- | --- | --- | --- | --- | --- | --- | --- | --- |
|  |  | **Gastrointestinal Cancer** | | | | **Total** | |  |
|  |  | **Yes** | | **No** | |  |  |  |
| **Monotherapy** | Statin | 365 | (1.3) | 28,631 | (98.7) | 28,996 | (84.1) | |
|  | Fibrates or bile acid | 11 | (1.2) | 923 | (98.8) | 934 | (2.7) | |
| **Combination therapy** | Statin+Ezetimibe | 25 | (1.0) | 2,520 | (99.0) | 2,545 | (7.4) | |
|  | Statin+Fibrates | 17 | (0.9) | 1,785 | (99.1) | 1,802 | (5.2) | |
|  | Statin+Ezetimibe or Fibrates | 0 | (0.0) | 211 | (100.0) | 211 | (0.6) | |
|  |  | 418 | (1.2) | 34,070 | (98.8) | 34,488 | (100.0) | |
